# *The H*AI*nich*: A multidisciplinary vision data-set for a better understanding of the forest ecosystem

**DOI:** 10.1038/s41597-023-02010-8

**Published:** 2023-03-27

**Authors:** Stefan Milz, Jana Wäldchen, Amin Abouee, Ashwanth A. Ravichandran, Peter Schall, Chris Hagen, John Borer, Benjamin Lewandowski, Hans-Christian Wittich, Patrick Mäder

**Affiliations:** 1 Technische Universität Ilmenau, Data-intensive Systems and Visualization, Ilmenau, 98693 Germany; 2 Spleenlab GmbH, Saalburg-Ebersdorf, 07929 Germany; 3 Max Planck Institute for Biogeochemistry, Biogeochemical Integration, Jena, 07745 Germany; 4 German Centre for Integrative Biodiversity Research (iDiv), Halle-Jena-Leipzig, Germany; 5 University of Göttingen, Silviculture and Forest Ecology of the Temperate Zones, Göttingen, 37077 Germany; 6 Friedrich Schiller University, Faculty of Biological Sciences, 07743 Jena, Germany

**Keywords:** Forestry, Civil engineering

## Abstract

We present a multidisciplinary forest ecosystem 3D perception dataset. The dataset was collected in the Hainich-Dün region in central Germany, which includes two dedicated areas, which are part of the Biodiversity Exploratories - a long term research platform for comparative and experimental biodiversity and ecosystem research. The dataset combines several disciplines, including computer science and robotics, biology, bio-geochemistry, and forestry science. We present results for common 3D perception tasks, including classification, depth estimation, localization, and path planning. We combine the full suite of modern perception sensors, including high-resolution fisheye cameras, 3D dense LiDAR, differential GPS, and an inertial measurement unit, with ecological metadata of the area, including stand age, diameter, exact 3D position, and species. The dataset consists of three hand held measurement series taken from sensors mounted on a UAV during each of three seasons: winter, spring, and early summer. This enables new research opportunities and paves the way for testing forest environment 3D perception tasks and mission set automation for robotics.

## Background & Summary

Accurate quantification of forest stand structure and dynamics is necessary to understand ecological processes and impacts of human activities. Forest site variables such as tree height, tree volume, and diameter at breast height (DBH), their spatial distribution and cover, are fundamental to ecosystem research and modeling of plant functional types, diversity, carbon balance, and ecophysiology^1–3^. For example, the biometric relationship between tree height and diameter is used to estimate biomass, which plays an important role in carbon cycling and climate modelling^4^. Ground-based forest inventories in which all trees in a forested stand are measured, are time-consuming, cost-intensive, and prone to human error^5^. To reduce the amount of field work required, foresters often use statistical and mathematical extrapolation based on measurements taken of sample circle plots. Typically the DBH and tree height are measured as they are strongly related to stem volume and above-ground biomass of the tree. Other tree parameters, such as the location, tree height, and height of the first living branch may also be recorded but are often not measured for every tree on sample plots because these measurements are labor-intensive^6^. Based on initial measurements, depending on the needs of research, foresters extrapolate the individual circles to the entire stand, which is liable to introduce large uncertainties^5^. Terrestrial laser scanning or Light Imaging Distance and Ranging (LiDAR)^7^ sensors have since simplified the acquisition process in some respects^8^.

These surveys must still be conducted by foresters and require a large amount of time and manpower, compared to a full automated measurement, which is still not possible. In recent years the use of automated remote sensing technologies for forest inventories has become the industry standard^9^. In particular, above canopy automated drone surveys have found widespread application however they still have limitations. Due to canopy occlusions it suffers from a lack of precision and is not capable of measuring the same attributes as ground based surveys. Automated ground based data acquisition with drones or ground rovers is currently an unsolved problem, as forest structures are extremely complex, unique, and congested spaces. This presents a huge challenge for autonomous robots, either air or ground based, to navigate and perceive their environment. However the benefit of introducing technologies that facilitate the automation of ground based high precision forestry surveys is so high that it is critical to closely study and solve the limiting technical factors.

In other disciplines with similar complex environments like automated driving, huge technical progress is being made both in commercialization and research. Similar data is collected and analyzed in real time to perceive and act autonomously. This progress is in large part due to the fact that engineers and researchers have access to many public datasets acquired in the relevant environments with the relevant sensors. The KITTI^10^, NuScenes^11^, and Woodscape^12^ and many more are important pioneering works with multi-modal perception data including LiDAR and camera measurements. Such a robotics dataset does not exist in the field of forestry science. We present the first intensive data set for robotics in forests including the full suite of modern perception sensors acquired in a forest setting. It is composed of a series of handheld measurements on a drone setup. The first non-automotive 360 vision dataset with fisheye and LiDAR capturing complex robotics forest scenarios combined with stand metadata captured by foresters, including stand age, diameter, exact 3D position, and species. Furthermore, the data set includes measurements at different points in time (winter, spring, summer), as the structures change significantly over the year. In this way, we want to advance research into automated inventory using both ground robots and most importantly drones.

## Methods

### Explanation of the forest areas

**Fig. 1:**
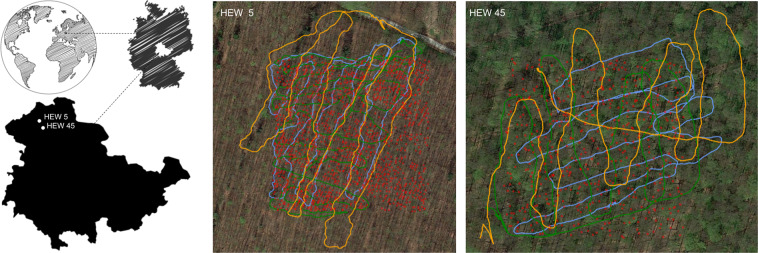
Study sites in the Hainich-Dün region in central Germany HEW5 (**a**) and HEW45 (**b**) are mapped with the handheld sensor setup at three different times (blue: run one - February, green: run two - March, orange: run three - May). Red dots indicate the ground truth tree positions from the meta studies.

**Fig. 2:**
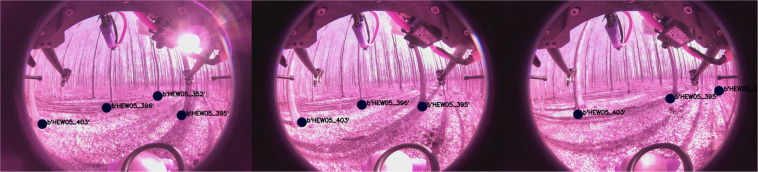
Projection of tree GPS coordinates onto image frames. This view is from the back camera.

**Fig. 3:**
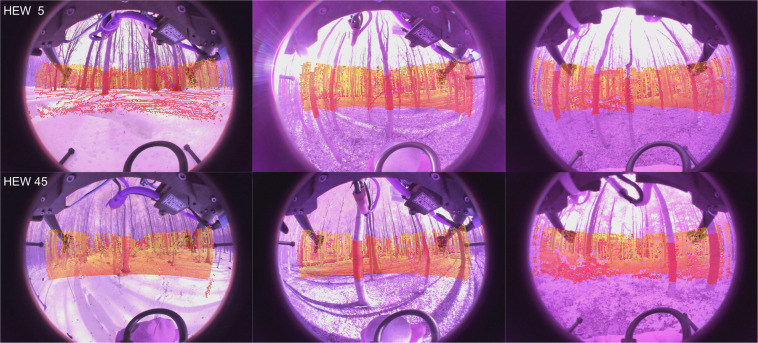
Sample images with the projected LiDAR ground truth (winter left, spring middle, summer right). The first row shows data from HEW 5 and the second row from HEW 45.

The study sites are located in the Hainich-Dün region in central Germany (see Fig. 1) and are part of so-called Biodiversity Exploratories, which are long-term research platforms to investigate the effects of varying land-use intensities on functional biodiversity response^13^. The forests are Beech (*Fagus sylvatica*) dominated admixed with *Fraxinus excelsior* and *Acer pseudoplatanus*. We selected two one hectare (100x100m) plots of single-layered stands (HEW5 and HEW45). A full forest inventory was carried out in winter 2020–2021, recording geographic position, species identity and diameter at breast height (DBH) of all trees with DBH > 7 cm using the FieldMap system^14^. For a subsample of trees distributed across the DBH gradient additionally tree height was measured. These subsample was used to estimate heights of all trees based on the Petterson function^15,16^. Tree volume (above bark) was subsequently estimated using height, DBH and species specific form factors^17^ An overview of the forest structure of both study areas is given in Table 1. An important aspect of planning a forest inventory is also the choice of an appropriate scanning date. For example, to see the difference between trees with foliage and without foliage, the images were repeated at three different times (see Figs. 2, 3) with accurate spatial ground truth (GPS and laser data).

**Table 1:** An overview of the stand characteristics of the study sites.

	HEW5	HEW45
Management	Age-class forest	Age-class forest
Stand Age (years)	97	41
Wood volume (m^3^ ha^1^)	434.9	286.1
Stand density (stems ha^1^)	431	1379
Mean diameter at breast height (cm)	27.1	16.3
Basal area (m h^−1^)	30.5	34.3
% Beech	96.0	96.1
other species	F. exc., A. pseud.	F. exc., A. pseud.
Mineral soil pH	5.0	7.1
Soil texture (clay/silt /sand %)	46.0 48.5 5.9	8.8 85.4 6.0
Standing dead wood (m^3^ ha^1^)	1.3	4.0

Data are given for all trees with a DBH > 7 cm.

### Sensors and data acquisition

**Fig. 4:**
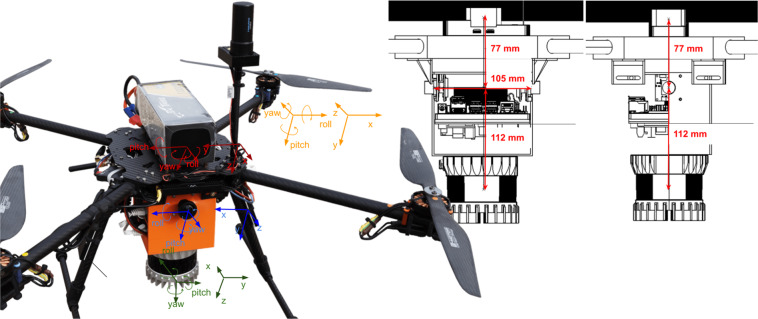
The quad-copter UAV setup deployed for the forest data measurements. Two fisheye cameras, a RTK GPS and a LiDAR such as a computing device (NVIDIA Jetson NX) are mounted on it.

A prototype UAV and 3D perception suite was used to acquire the sensor dataset. The configuration consists of an Ouster OS1-64 LiDAR sensor, two eCon e-CAM50 CUNX/NANO 5MP cameras with Lensagon 190° BF10M14522S118 (no IR-Filter), and a Holybro F9P RTK GPS. The sensor setup was mounted on a handheld Tarot Ironman 650FY drone. The experimental configuration is illustrated and annotated in Fig. 4. The embedded computing device is a Nvidia Jetson NX developer kit. ROS^18^ Melodic middleware was used to communicate with all sensors. The Ouster LiDAR and the cameras have their own ROS drivers. The entire setup was powered by a 6 S LiPo Battery.

### Calibration and correction

**Fig. 5:**
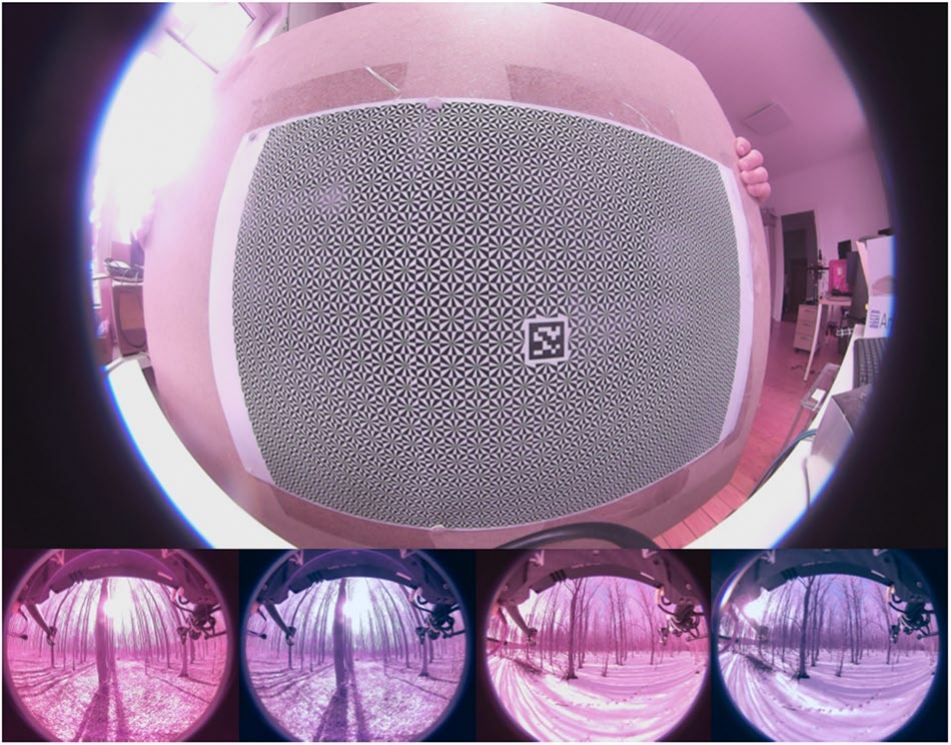
Calibration pattern (top) and color correction (bottom).

**Fig. 6:**
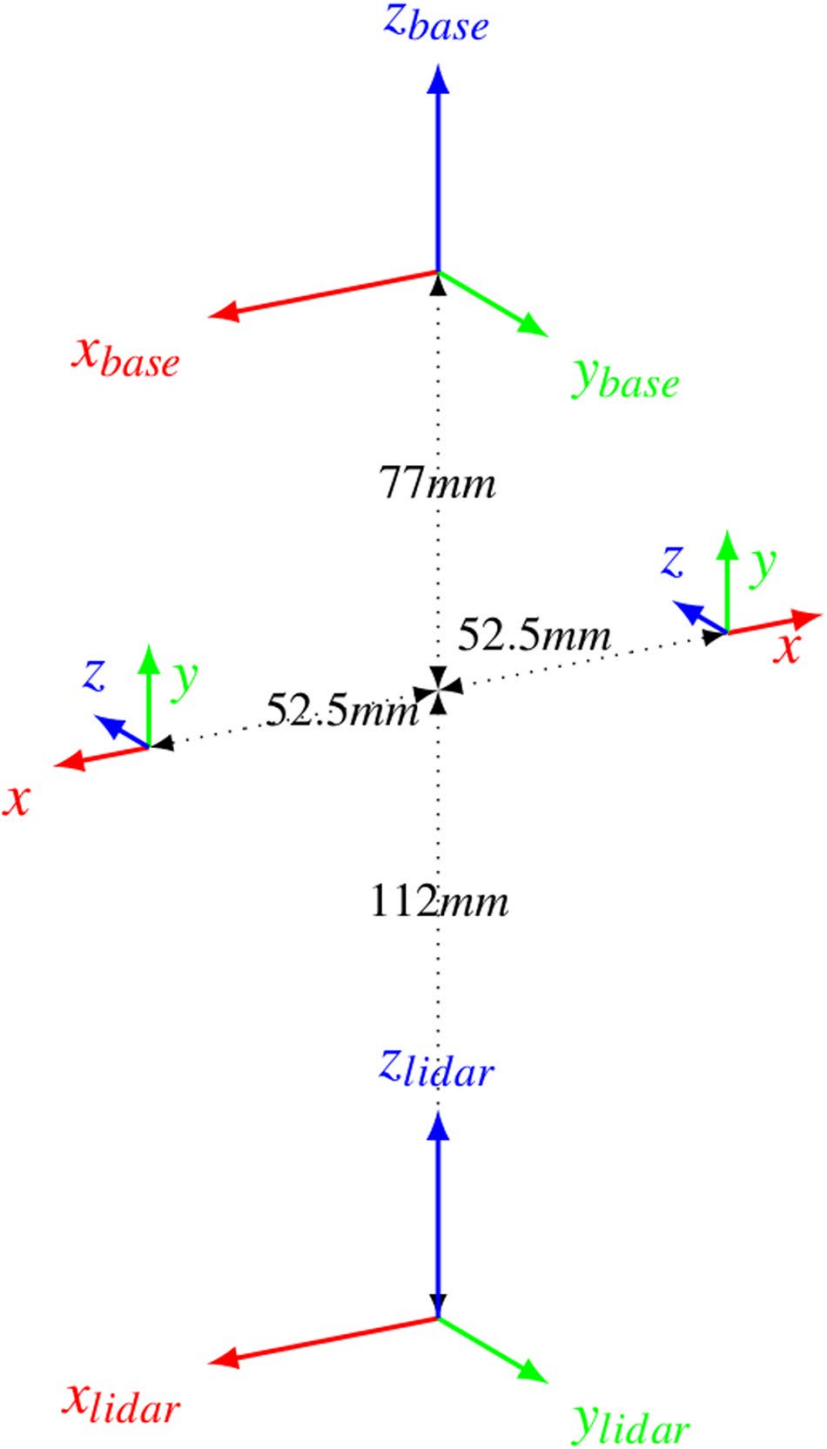
Extrinsics.

The two fisheye cameras were calibrated using Puzzlepaint camera calibration^19^. The calibration pattern used is an A3 size Puzzlepaint pattern with 16 star segments, each square of length 1.2 cm. In the pattern center is an April-Tag of size 2 cm. The Puzzlepaint pattern can also be seen in Fig. 5. The calibration pattern is provided in calib_pattern.pdf and calib_pattern_config.yaml. It contains the configuration for the pattern. Image data was acquired using a lens with no IR-filter, we provide a color-correction module listed in Table 2 and shown in Fig. 5. Extrinsic calibrations of all sensors are shown in Fig. 6 (see also Fig. 4).

**Table 2:** Structure of the provided files.

## Data Records

The dataset is available under Dryad^20^ and Zenodo^21^. In both repositories the data is stored and zipped into a single file named forest.zip with a instruction file called README.txt. The data directory structure and all data content is shown in Table 2. A calibration sub-folder includes: the calibration pattern, the config and the calibration result (calib_pattern.pdf, puzzlepaint_config.txt, calib_pattern_config_yaml) such as some drawings for a better understanding of the extrinsic configuration (sub-folder Extrinsic). The metadata is stored in the sub-directory meta_data in Ground_Truth.xlsx. This file includes the manual acquired data by foresters (see Table 1) for all 1967 trees in the areas with the following fields: *EP−area, Lat, Lon, GK*_*R*_*, GK*_*H*_*, X*_*m*_*, Y*_*m*_*, Z*_*m*_*, ID, date, DBH*_*m*_*m, species, multistem, brakewood, year*. A sub-directory Location includes optional geo-information of the areas. All raw sensor data are provided as rosbags (e.g. h1f1r1.bag, see ROS^18^). Three recordings were done during summer, spring and winter for HEW5 and HEW45 (forest_1 and forest_2). The naming, e.g. abc.bag, with a being either h1 (winter), h2 (spring), h3 (summer) and b being f1 for HEW5 or f2 for HEW45. c is an id (r1 or r2). Due to space limitations all meta data (including calibration) is published under Dryad^20^ and all raw sensor recordings under Zenodo^21^. Both repositories needs to be used for the full data-set.

### Ground truth position

RTK GPS was used to determine the global position of the UAV. GPS coordinates and IMU measurements are fused together with an Extended Kalman Filter to estimate ground truth 3D odometry. It is recorded at a frequency of 10 Hz. Odometry ground truth is shown in Fig. 1. The ground truth odometry is provided for each rosbag (gt_odom.txt). LiDAR data, shown in Fig. 3, provides the ground truth for depth estimation, and the extrinsic calibration provides a ground truth for object detection.

## Technical Validation

The dataset is the first 360 vision dataset using fisheye camera imagery with LiDAR ground truth in the non-automotive sector (forestry) in combination with geo-localization sensors (see Fig. 1). Several measurements were performed at different times of the year and therefore unique temporal environmental information is also available. Furthermore, we publish manually but equally geolocated tree data by the foresters (see Table 1). For validation, we plotted the georeferenced pose data of all acquisition runs (several year times) in a common coordinate system along with the manually collected metadata. The results are conclusive and confirm the integrity of the data (see Fig. 1). Both sides HEW5 and HEW45 are clearly visible, overlap in all raw data, meta data and satellite imagery.

**Fig. 7:**
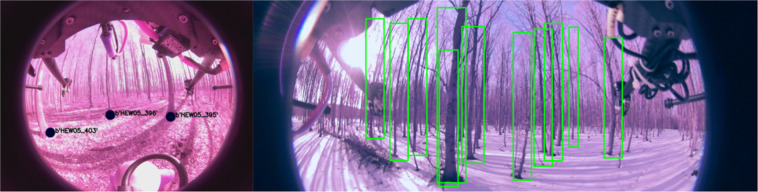
Automated bounding box extraction (right) for the fisheye rgb camera data object detection using the extrinsic calibration (left) which can be combined with the meta information like diameter or specie.

**Fig. 8:**
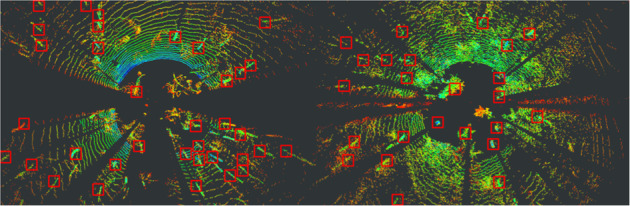
Exemplary bounding box extraction for a LiDAR birds eye view projection (winter sample left, summer sample right) using standard width and height and the extrinsic localization of the meta data.

**Fig. 9:**
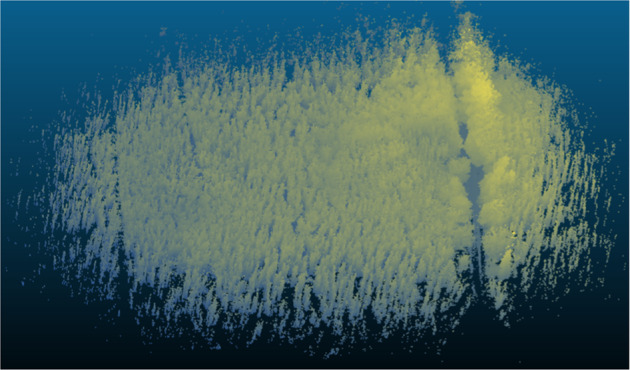
High resolution mapping result using the accumulated lidar data of h1f1r1.

In the methods section (intrinsics see Fig. 5, extrinsics see Figs. 4, 6) our calibration of the sensors is explained along with our time synchronization. The calibration of these sensors is the base of every perception task. First we validate, intrinsic and extrinsic calibration including time synchronisation with the aid of LiDAR to image projection. In the optimal case, structures (e.g. trees) overlap perfectly in the entire projection image, i.e. identical structures are displayed by the LiDAR and RGB image at the same spatial location. In Fig. 3, we see perfect qualitative results for all acquisition runs an both cameras. We see an optimal overlap, any error in time synchronization or spatial calibration would result in a shifted projection. Furthermore, we have projected the manually measured 3D tree points into the camera images by means of geo-referencing (see Fig. 2) and calibration. The manually captured trees are perfectly visible in the camera image. This calibration and geo-referencing is the basis for automated annotation, i.e. the mapping of existing metadata in the spatial captured sensor data. This is shown in the Figs. 7, 8 for Camera and LiDAR data. Tree structures are clearly visible within the defined bounding boxes. The integrity of the inertial and GPS data was confirmed in Fig. 9. All LiDAR frames (h1lr1) were accumulated by positional data and a clear 3D map of the forest was obtained.

## Usage Notes

This new dataset that for the first time combines multi-modal sensor data collection for autonomous vehicles and 3D perception with forest science and opens a broad array of bleeding edge research interests for scientists in the areas of interest presented here. Therefore, as Usage Notes, we demonstrate exemplary four experiments in perception for robotics in forestry: object detection, depth estimation, localization or path planning. These experiments show that this data set can be used to advance the relevant research interests. For the reason of usage, the data set is released with benchmark metrics with the intention that these will be built upon by the research community later on.

### Object detection and classification

Object recognition and classification is a critical ability for autonomous vehicles. Using this technology trees can be automatically located and classified using both camera and LiDAR. Recent state-of-the-art methods perform object detection on camera and LiDAR including SSD^22^, Yolo^23^, Complex Yolo^24^. The presented dataset’s metadata, extrinsics, and ground truth odometry allow for the labeling of forest features at scale, offering for the first time training data for forest environment recognition and classification tasks. Numerous characteristics such as diameter or age of the tree can be directly based on image or LiDAR measurements. Figure 7 shows example camera images and Fig. 8 LiDAR data.

### Monocular depth estimation

**Fig. 10:**
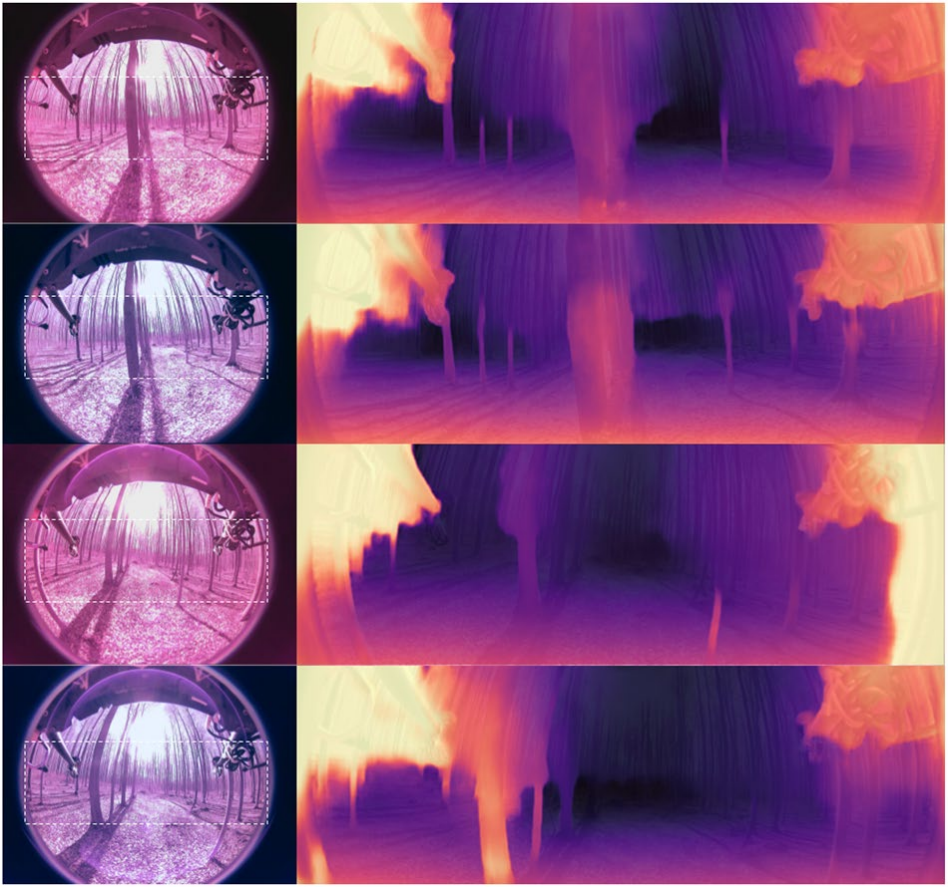
Qualitative results using SOTA Monocular Depth estimation. The left column shows four samples on the spring collection of our data-set (two uncorrected IR images, two color corrected images). We cropped the area of the image as input for the used approach Monodepth^25^. These results are shown as overlay depth maps on the right column. The initial results are promising, paving the way towards autonomous forest navigation on cameras with 360 degree field of view.

The challenge of predicting a dense depth map from a single RGB image is known as “single image depth estimation”. Here we provide the first non-automotive public fisheye camera 360-degree FOV dataset with LiDAR ground truth (Fig. 3). This enables further research as provided by^25^ on monocular depth estimation or^26^ on fisheye depth estimation. A baseline experiment using the Monodepth2 approach of^25^ pretrained on automotive data (KITTI^10^) on a crop of the a set of color corrected and uncorrected images showed potential, sample results are shown in Fig. 10. We ran the pretrained model on 100 sample test images, two times (with and without color correction) and calculated the sparse RSME as proposed by^26^ using the LiDAR ground truth (Fig. 3) capped at 30 m. We achieved an RSME of higher 5 for the raw data and around 4 for the corrected images, which is promising and it shows that the color correction has a positive impact Table 3.

**Table 3:** Total number of messages in each dataset.

Dataset	Duration	Poses	Camera	Lidar
h1f1r1	1010 s	10110	6428	10109
h1f2r2	965 s	9658	6037	9658
h2f1r1	1380 s	13810	34629	13811
h2f2r2	599	6000	16652	6001
h3f1r2	999	9671	25903	10000
h3f2r2	641	0	17878	6420

### 3D Mapping and localization

**Fig. 11:**
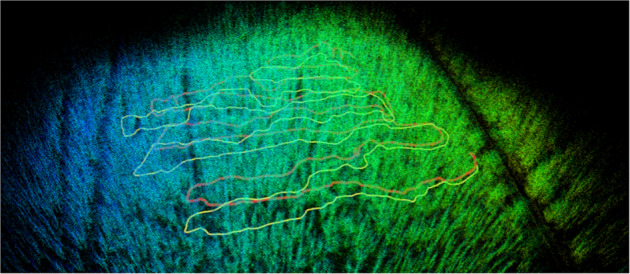
**Qualitative results** from our baseline experiments. The image shows the trajectories of the A-LOAM algorithm (yellow) and GPS ground truth (red) such as the accumulated map (h1f1r1).

3D Mapping (qualitative sample in Fig. 9) and Localisation is one of the most important tasks, as it calculates the position and a map simultaneously and therefore could be used for analytical tasks like biomass calculation or tree segmentation in 3D as the position calculation of the robot for automated navigation. We present an extensive evaluation study for the pose error verification and qualitative results for the mapping, shown in Fig. 11. The pose error between the ground truth *Q*_1:*n*_ ∈ *SE*(3) and estimated trajectories *P*_1:*n*_ ∈ *SE*(3) is quantified with the absolute trajectory error (ATE), and relative pose error (RPE) metrics^10,27^. ATE measures the global consistency of a trajectory. It is determined by comparing the absolute distances between the estimated and the ground truth trajectory.As both trajectories might be defined in any coordinate frame, they must first be aligned. In this evaluation, the Umeyama^28^ alignment was used as a pre-processing step to find the 3D rigid-body transformation *S*, that maps the estimation *P*_1:*n*_ onto the groundtruth *Q*_1:*n*_. The ATE at timestep *i* can be calculated as

$$AT{E}_{i}\,:\,={Q}_{i}\ominus S{P}_{i}={Q}_{i}^{-1}S{P}_{i}$$

The root mean square error (RMSE) is usually used for both translational and rotational parts of ATE separately, and serves as the quality metric.

$$RMSE(AT{E}_{pos}):\,=\sqrt{\frac{1}{n}\mathop{\sum }\limits_{i=1}^{n}{\parallel pos(AT{E}_{i})\parallel }^{2}}\quad \quad \quad RMSE(AT{E}_{rot}):\,=\sqrt{\frac{1}{n}\mathop{\sum }\limits_{i=1}^{n}{\parallel \angle (AT{E}_{i})\parallel }^{2}}$$

where ⊖ represents the inverse compositional operator^29^ and ∠(․) is the rotation angle in degrees. The RPE measures the local accuracy of a SLAM trajectory over a fixed time interval Δ. The RPE at timestep *i* can be calculated as

$$RP{E}_{i}\,:\,=({Q}_{i}\ominus {Q}_{i+\Delta })\ominus ({P}_{i}\ominus {P}_{i+\Delta })={({Q}_{i}^{-1}{Q}_{i+\Delta })}^{-1}({P}_{i}^{-1}{P}_{i+\Delta })$$

**Fig. 12:**
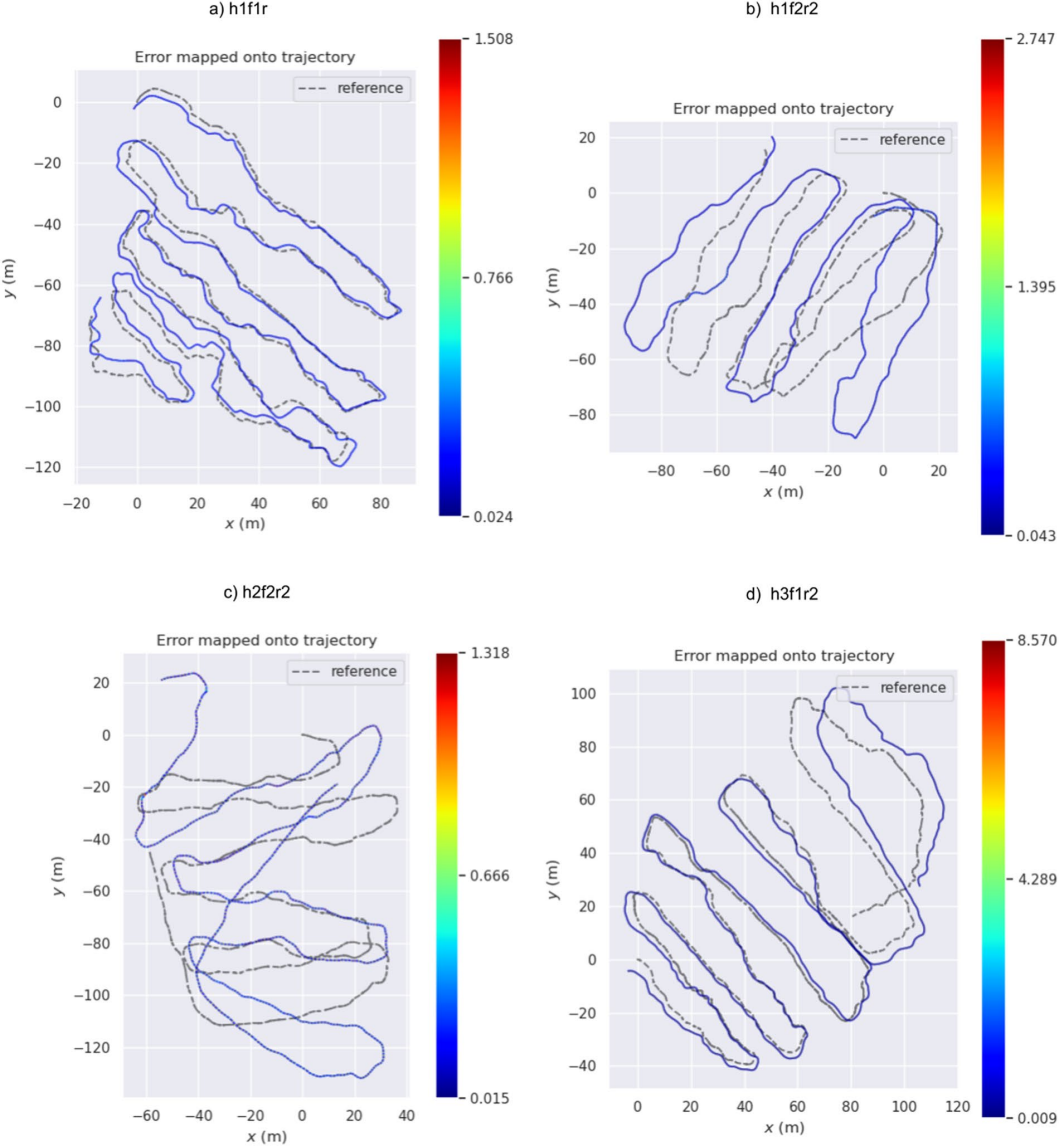
Relative Pose Error (RPE) for our baseline method (ALOAM). The error represents the full transformation error between the ground-truth and estimated trajectories. The dashed gray illustrates the ground-truth while the colored is estimated by ALOAM.

In this evaluation, we set Δ = 1 to perform the RPE for all consecutive frames (visual odometry). As experiment for evaluation, we run A-LOAM (see code availability). A-LOAM is an optimized version of LOAM^30^ which is one of the state-of-the-art algorithm in Lidar localization that can identify the pose and the map of the environment in real-time. The algorithm perform precisely in our dataset, results are presented in Table 4 and Fig. 12.

**Table 4:** A-LOAM results.

		ATE		RPE	
		pos (m)	rot(deg)	pos(m)	rot(deg)
HEW5	h1f1r1	3.867590	174.526263	0.157067	4.012467
	h1f2r2	12.980364	177.575609	0.218814	5.403690
HEW45	h2f2r2	32.752117	175.992315	0.367303	4.803951
	h3f1r2	15.751300	175.474383	0.279253	4.346198

### Path planning

**Fig. 13:**
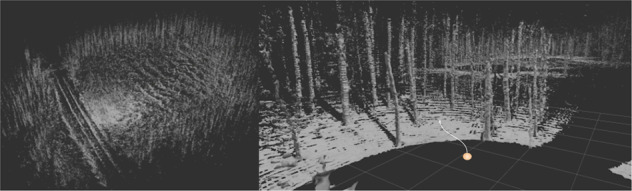
**Qualitative results** of TSDF based map generation using Voxblox^31^. The figure shows the result of (h1f1r1). The left a global view and the right zoomed near-field. Trees and paves are clearly visible. The representation can be directly used for path planning.

Autonomous robotic platforms build an internal representation of the observed world to plan and execute motion tasks within. This observation is real-time critical and needed to be handled with an high update rate. Accumulated or mapped point-clouds, as shown in Fig. 11, are too complex to be used for real-time path planning. Representations which use LiDAR as input, like Signed Distance Function (SDF) maps, are fast and have low overhead. In this section we use the Voxblox algorithm, proposed by^31^ using Volumetric Mapping using Truncated Signed Distance Fields (TSDF), to generate a map for planning. We perform this experiment on hlfr1 and generated competent maps for path planning in the complicated forest environment. The results are shown in Fig. 13. Trees as well as paths are clearly separated and are easy to recognize visually. Furthermore they can readily be identified by path planning algorithms and used to plan collision free navigation trajectories. This shows that our data is suited for path planning research in forest environments, bringing research in this area a decisive step forward.

## Data Availability

No explicit code has been developed in conjunction with the data-set. However, several resources are necessary to use the data. For replaying, using and developing the raw data and to perform sensor fusion such as individual tasks, it is recommended to install ROS^18^ (robot operating system) Melodic middleware documented at http://wiki.ros.org/Documentation with all necessary download links. For our presented camera calibration, which is the base for several tasks, Puzzlepaint camera calibration^19^ is used with the codebase under https://github.com/puzzlepaint/camera_calibration. For the depth estimation in this research-work we simply used the self-supervised approach from Godard *et. al*^25^. The software is available under https://github.com/nianticlabs/monodepth2. The localization algorithm A-LOAM from Zhang *et. al*^30^, available at https://github.com/HKUST-Aerial-Robotics/A-LOAM, was used for the localization task. We used Voxblox^31^ for path planning (https://github.com/ethz-asl/voxblox).
